# Hemangioma of the Atherosclerotic Changed Aortic Valve

**DOI:** 10.1155/2019/7916298

**Published:** 2019-03-07

**Authors:** A. van Broekhoven, P. A. J. Krijnen, H. W. M. Niessen, A. B. A. Vonk

**Affiliations:** ^1^Department of Pathology, Amsterdam UMC, Vrije Universiteit, Amsterdam, Netherlands; ^2^Amsterdam Cardiovascular Sciences, Amsterdam, Netherlands; ^3^Department of Cardiac Surgery, Amsterdam UMC, Vrije Universiteit, Amsterdam, Netherlands

## Abstract

The incidence of heart valve hemangioma is very low and is mostly observed in the mitral and tricuspid valve. In 2006, two cases of aortic valve hemangioma were reported for the first time, including one with calcifying aortic valve stenosis. We now present a case of aortic valve hemangioma in a patient suffering from aortic valve insufficiency combined with atherosclerotic thickening.

## 1. Introduction

Primary benign tumors of the heart are rare, accounting for 72% of all primary cardiac tumors [1]. Of these benign tumors, only 5-10% consist of hemangiomas. Valvular hemangiomas are even less frequent [2, 3]. Most previous case reports described valve hemangiomas observed in the mitral or tricuspid valve [4–14]. To the best of our knowledge, only two aortic valve hemangiomas have been reported to date, including one hemangioma observed in a calcified stenotic aortic valve [15, 16]. In this report, we present a new case of hemangioma which was unexpectedly detected in an aortic valve with atherosclerotic changes after pathological examination.

## 2. Case Presentation

A 74-year-old man was referred to our hospital with severe aortic valve insufficiency and left ventricle dilatation. His past medical history showed hypercholesterolemia and a recent episode of de novo atrial flutter which was treated with electrical cardioversion. His medication included atorvastatin, nebivolol, and dabigatran. He stopped smoking 40 years ago and uses 14 units of alcohol per week.

The patient's vital signs were normal. Physical examination revealed normal pulmonary, abdominal, and neurological functions. Except for a diastolic murmur at the right lower sternal border, cardiovascular examination revealed normal heart sounds. Coronary angiography did not show significant coronary artery disease. Transthoracic echocardiography revealed a severe aortic valve insufficiency with a somewhat impaired function of the left ventricle. Cardiac MRI showed severe aortic valve insufficiency combined with a dilated aortic root (46 mm) and a severely dilated left ventricle with an ejection fraction of 50%. Aortic valve replacement with a biological device (Edwards Lifesciences Perimount 27 mm) was performed. No complications occurred during surgery. Except for a ventral pneumothorax, the patient's recovery was uneventful. He was discharged in good condition to the cardiology nursing ward of a peripheral hospital on postoperative day 4. At two weeks follow-up, the patient was doing well. He did not experience any cardiac complaints. For further recovery, the patient was referred back to the referring cardiology department. After 6 weeks, the patient was reevaluated for his recovery at the policlinic of cardiothoracic surgery, where no abnormal findings were observed during physical examination.

**Figure 1:**
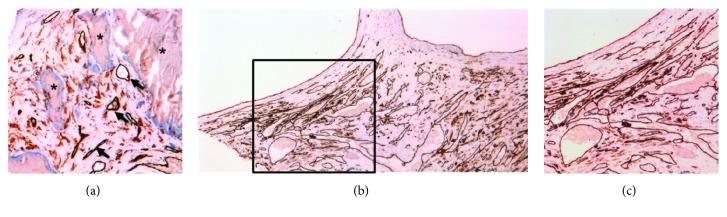
Reactive proliferation of blood vessels. (a) Reactive proliferation of dense blood vessels (arrows) surrounding calcified areas (asterisk) visualised by CD31 staining. Magnification 50x. (b) Overview of aortic valve hemangioma stained for CD31, showing an agglomerate of thin-walled vessels. Magnification 25x. (c) Detailed view of aortic valve hemangioma stained for CD31. Magnification 50x.

**Figure 2:**
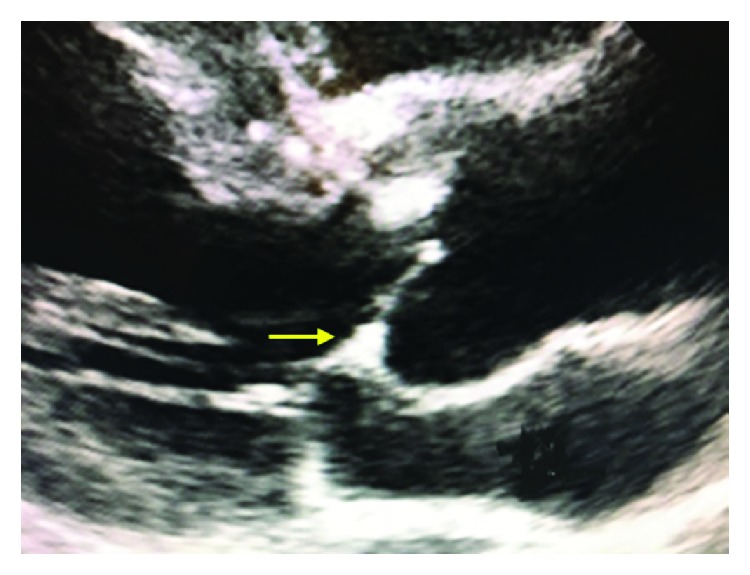
Preoperative transthoracic echocardiography. Apart from a slightly thickened aortic valve (arrow), no clear abnormalities indicative for a hemangioma could be observed.

The excised aortic valve was tricuspid and measured 3.5 × 1.3 × 0.3 cm. The cusps were partially calcified. Microscopic examination of cross-sections of the aortic valve leaflets showed areas of calcification with focal ceroid, as can be found in the atherosclerotic valves [17, 18]. In between the calcification areas, a few reactive small thin-walled blood vessels were found (Figure 1(a)). Next to calcification, fibrosis and mucoid degeneration was also observed (not shown). In the periphery of the valve, an agglomerate of thin walled, often dilated, blood vessels was observed (Figures 1(b) and 1(c)). These vessels stained positive for CD31 and were negative for D2-40 (staining lymphatic vessels). The vessels were predominantly negative for SMA (not shown). These findings supported the diagnosis of hemangioma of the aortic valve. In retrospect, no clear abnormalities indicative for a hemangioma in the aortic valve were seen in preoperative TTE. The aortic valve did appear slightly thickened, which was assumed to be due to atherosclerotic changes (Figure 2).

## 3. Discussion

Hemangiomas of the cardiac valves are exceptional, especially in the aortic valves [15, 16].

We now describe the finding of a hemangioma in the atherosclerotic aortic valve. Previously, Val-Bernal et al. also described a hemangioma in a calcified aortic valve [16], while Vivirito et al. described a hemangioma in a nondegenerated valve, indicating that hemangiomas also occur independent of atherosclerosis. It is known that in atherosclerotic aortic valves, reactive vessel wall proliferation occurs. Normally, these are small solitary vessels. In contrast, in hemangioma, an agglomerate of multiple thin-walled and mostly dilated vessels occurs, excluding a reactive vessel wall proliferation as demonstrated in our case.

The clinical presentation of cardiac hemangiomas is variable and depends on multiple factors including location, size, growth rate, sex, and patient's age. Heart valve hemangiomas may present with palpitations and syncope, exertional dyspnea, heart failure due to hemodynamic instability, and atypical chest pain [2, 6, 8, 10, 14, 19–21]. In our patient, it is likely that the hemangioma of the aortic valve contributed to the development of aortic valve regurgitation. As most patients with heart valve hemangiomas remain asymptomatic (Edwards [2]), it cannot be precluded that aortic valve hemangiomas are more common than observed, since they may not cause clinically relevant valve dysfunction.

Cardiac hemangiomas can be detected by transthoracic echocardiography (TTE), transesophageal echocardiography (TEE), and cardiac CT or MRI scan [22]. To differentiate hemangiomas from other cardiac tumors, a three-dimensional TTE (3D TTE) may be more precisely related to so-called echolucencies [8, 9]. However, the golden standard for diagnosis of most cardiac hemangiomas is histological examination upon surgical excision [8, 9]. In our case, no signs indicative for the presence of an aortic valve hemangioma on preoperative TTE and cardiac MRI were seen, even in retrospect. The thickening of the aortic valve that was observed was assumed to be due to atherosclerosis. However, we did not perform 3D TTE in our patient. Previous case reports regarding aortic valve hemangioma also did not detect the hemangioma using TTE [15, 16].

In general, cardiac hemangiomas are classified as benign tumors and lack the ability to metastasize. Their growth patterns are unpredictable and range from dormancy to accelerated growth or to spontaneous regression [3, 8, 23–25]. To the best of our knowledge, the growth pattern of valvular hemangiomas specifically is unknown, since all the previous documented cases were obtained from excised heart valves and therefore the natural course of these hemangiomas was not studied. In one case of a surgically excised histiocytoid hemangioma of the left atrium, an angiosarcoma was found 7 years after surgery, which supports the recommendation of regular follow-up by echocardiography of cardiac hemangiomas [8, 11, 26]. Though, as far as we are aware, in all patients with heart valve hemangiomas described so far, no signs of recurrence of the hemangioma have been observed [27] nor malignant degeneration of heart valve hemangiomas or any other complications have been described upon excision [6, 27].

In our case, the patient was reevaluated six weeks after aortic valve replacement by the cardiothoracic surgeon. No abnormal findings were observed during the physical examination. Therefore, upon en bloc removal of the aortic valve containing the hemangioma, there are no specific consequences regarding surgical follow-up for our patient. Instead, regular check-ups need to be aimed at evaluating the prosthetic valve and the patient's status after severe aortic valve insufficiency.

In conclusion, we present a hemangioma localized in an atherosclerotic aortic valve [15, 16]. Several imaging techniques, including TTE and cardiac MRI, did not reveal the existence of a tumor mass in our patient, indicating the importance of pathological examination of excised heart valves.
